# Preparation and Fractionation of Xenopus laevis Egg Extracts

**DOI:** 10.3791/891

**Published:** 2008-08-27

**Authors:** Marie K. Cross, Maureen Powers

**Affiliations:** Department of Cell Biology, Emory University

## Abstract

Crude and fractionated Xenopus egg extracts can be used to provide ingredients for reconstituting cellular processes for morphological and biochemical analysis. Egg lysis and differential centrifugation are used to prepare the crude extract which in turn in used to prepare fractionated extracts and light membrane preparations.

**Figure:**
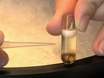


## Protocol

The complete text protocol for this experimental approach is available in Current Protocols in Cell Biology.

